# Dystroglycanopathy: From Elucidation of Molecular and Pathological Mechanisms to Development of Treatment Methods

**DOI:** 10.3390/ijms222313162

**Published:** 2021-12-06

**Authors:** Motoi Kanagawa

**Affiliations:** Department of Cell Biology and Molecular Medicine, Graduate School of Medicine, Ehime University, 454 Shitsukawa, Toon 791-0295, Ehime, Japan; kanagawa.motoi.fa@ehime-u.ac.jp; Tel.: +81-89-960-5248

**Keywords:** muscular dystrophy, glycosylation, dystroglycan, therapy, model mouse, ribitol-phosphate

## Abstract

Dystroglycanopathy is a collective term referring to muscular dystrophies with abnormal glycosylation of dystroglycan. At least 18 causative genes of dystroglycanopathy have been identified, and its clinical symptoms are diverse, ranging from severe congenital to adult-onset limb-girdle types. Moreover, some cases are associated with symptoms involving the central nervous system. In the 2010s, the structure of sugar chains involved in the onset of dystroglycanopathy and the functions of its causative gene products began to be identified as if they were filling the missing pieces of a jigsaw puzzle. In parallel with these discoveries, various dystroglycanopathy model mice had been created, which led to the elucidation of its pathological mechanisms. Then, treatment strategies based on the molecular basis of glycosylation began to be proposed after the latter half of the 2010s. This review briefly explains the sugar chain structure of dystroglycan and the functions of the causative gene products of dystroglycanopathy, followed by introducing the pathological mechanisms involved as revealed from analyses of dystroglycanopathy model mice. Finally, potential therapeutic approaches based on the pathological mechanisms involved are discussed.

## 1. Introduction

Muscular dystrophy is a group of genetic disorders with progressive muscle weakness, and >50 causative genes have been identified (www.musclegenetable.fr, accessed on 1 November 2021). Dystroglycanopathy (DGpathy) is a collective term referring to muscular dystrophies with abnormal glycosylation of dystroglycan (DG). DG was first identified as a glycoprotein that interacts with dystrophin, the causative gene product of Duchenne muscular dystrophy, in skeletal muscle [1]. Once translated, DG is cleaved into α and β subunits, both of which are expressed in muscle cell membranes, interacting with each other in a non-covalent fashion. αDG is an extracellular subunit containing an exceedingly large number of sugar chains, and its binding to basement membrane and synaptic proteins is mediated by the sugar chains. Its known ligands include molecules containing laminin G-domain, such as laminin and agrin [2]. On the other hand, βDG is a transmembrane protein that binds to αDG on the cell surface and to dystrophin intracellularly. Since dystrophin binds to the actin cytoskeleton, DG plays a role in connecting the basement membrane and cytoskeleton across the cell membrane.

In the early 2000s, αDG sugar chain abnormalities and loss of laminin-binding activity were reported in specimens of patients with Walker–Warburg syndrome (WWS), muscle–eye–brain disease (MEB), Fukuyama congenital muscular dystrophy (FCMD), congenital muscular dystrophy 1C, and limb-girdle muscular dystrophy (LGMD) 2I (alternatively called LGMDR9) [3,4,5,6]. Because DG and dystrophin are normally expressed in cell membranes in the tissues of these patients, αDG sugar chain abnormalities are thought to disrupt the coordination between the basement membrane and the cytoskeleton [7]. Since then, cases showing similar sugar chain abnormalities have been reported, which led to the establishment of the disease concept of DGpathy. To date, at least 18 causative genes of DGpathy have been identified [8] (Table 1).

DGpathy exhibit a broad clinical spectrum, ranging from severe congenital muscular dystrophies, such as WWS and FCMD, to mild, adult-onset LGMD [9]. This is likely due to the effects of mutations involving the functions of gene products (enzymatic activity), rather than to variation in causative genes. In addition, one of the characteristics of DGpathy is the involvement of central nervous system disorders, such as malformation of the brain (type II lissencephaly) and mental retardation. Brain lesions in DGpathy have also shown a wide range of clinical symptoms, ranging from severe malformation of the brain to lesions with only mental retardation without structural abnormalities [10,11]. Furthermore, cases with heart failure and eye symptoms have also been observed. However, there are currently no effective treatment methods for any of these symptoms.

## 2. Sugar Chain Structure of DG and Functions of Causative Genes of DGpathy

**Figure 1:**
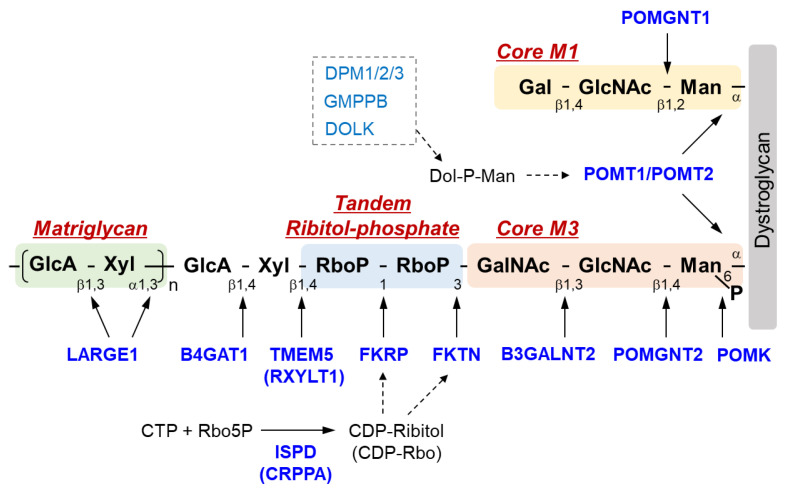
Overview of DG sugar chain structure and modifying enzymes. Man, mannose; GlcNAc, *N*-acetylglucosamine; GalNAc, *N*-acetylgalactosamine; RboP, ribitol phosphate; Xyl, xylose; GlcA, glucronic acid; Gal, galactose; Rbo5P, ribitol-5-phosphate; POMK, Protein *O*-mannose kinase; POMGNT, Protein *O*-mannose *N*-acetylglucosaminyltransferase; B3GALNT2, β1,3-*N*-acetylgalactosaminyltransferase 2; TMEM 5, Transmembrane protein 5; RXYLT1, ribitol-5-phosphate xylosyltransferase 1; B4GAT1, β-1,4-glucuronyltransferase 1; LARGE1, like-acetylglucosaminyltransferase 1/ LARGE xylosyl- and glucuronyltransferase 1; POMT, protein *O*-mannosyl-transferase; FKTN, fukutin; FKRP, Fukutin-related protein; ISPD, isoprenoid synthase domain-containing protein; CRPPA, CDP-ribitol pyrophosphorylase A.

The sugar chain structure involved in the ligand binding activity of DG and the enzymes involved in its biosynthesis are shown in Figure 1. *O*-Mannosyl glycosylation is required for the binding of DG to its ligands, and some of the causative genes of DGpathy encode enzymes involved in the biosynthesis of sugar chains named Core M1 (galactose-β1,4-*N*-acetylglucosamine-β1,2-mannose-; Gal-β1,4-GlcNAc-β1,2-Man-) and Core M3 (*N*-acetylgalactosamine-β1,3-*N*-acetylglucosamine-β1,4-mannose-; GalNAc-β1,3-GlcNAc-β1,4-Man-). The protein-*O*-mannose transferase (POMT) 1-POMT2 complex functions to transfer a mannose moiety to serine/threonine residues of DG [12]. Because the POMT1-POMT2 complex uses dolichol phosphate mannose (Dol-P-Man) as a donor substrate, mutations in the genes (*GMPPB*, *DPM1/2/3*, and *DOLK*) that encode enzymes involved in its biosynthesis have been reported to cause DGpathy (Table 1) [8]. The sugar chains of Core M1 are extended by protein *O*-mannose *N*-acetylglucosaminyltransferase (POMGNT1) [13], while those of Core M3 are extended by POMGNT2 and β1,3-*N*-acetylgalactosaminyltransferase 2 (B3GALNT2) [14].

After the Core M3 trisaccharides have been modified, mannose is phosphorylated by protein *O*-mannose kinase (POMK) [14]. On the non-reducing terminal side of Core M3, two tandemly-connected sugar alcohol phosphates, called ribitol-phosphates, are modified (tandem ribitol-phosphate) [15]. This modification is carried out by two ribitol-phosphate transferases, fukutin (FKTN) and fukutin-related protein (FKRP); the first ribitol-phosphate is modified by fukutin, while the second is modified by FKRP. The donor substrate for ribitol-phosphate is CDP-ribitol (CDP-Rbo), which is produced from ribitol-5-phosphate (Rbo5P) and CTP by the enzymatic action of isoprenoid synthase domain-containing protein (ISPD) [15,16,17]. Additionally, the names CDP-ribitol pyrophosphorylase A (CRPPA) and D-ribitol-5-phosphate cytidylyltransferase have also been proposed for ISPD based on its enzymatic activity. FCMD, which is caused by mutations in *FKTN*, is the predominant form of DGpathy in Japan, and LGMD2I, which is caused by mutations in *FKRP*, is the most frequent form of DGpathy in the United States and Europe. DGpathy patients with *ISPD* mutations have been often reported (see review [2]). Together, diseases caused by defects in ribitol-phosphate modification can account for the majority of DGpathy.

The end of tandem ribitol-phosphate is followed by xylose and glucuronic acid modified by ribitol-5-phosphate xylosyltransferase 1 (RXYLT1; previously transmembrane protein 5, TMEM5) and β1,4-glucuronyltransferase 1 (B4GAT1) [18,19]. This is then extended with repeating unit of disaccharides consisting of xylose and glucuronic acid, which is formed by LARGE1 (like-acetylglucosaminyltransferase 1/LARGE xylosyl- and glucuronyltransferase 1) [20]. As this repeat structure functions as a binding site for matrix ligands [21,22], it is also called matriglycan. The loss of function of any of the causative genes of DGpathy results in the termination of sugar chains synthesis, preventing the formation of matriglycan. Thus, abnormalities in any of the genes result in matriglycan deficiency. POMGNT1, which is a modifying enzyme of Core M1, is thought to be involved in matriglycan modification by binding to Core M3 and fukutin [23]. Mannosyl phosphorylation of Core M3 is required for ribitol-phosphate modification by FKRP and for matriglycan extension by LARGE1 [24,25]. Thus, many enzymes are required for DG glycosylation, and modification specificity may be created by a series of enzymatic reactions that proceed in an extremely orderly manner.

Recently, cases of congenital disorders of glycosylation (CDG) exhibiting reduced DG glycosylation have been reported. In a case with a mutation in mannose-phosphate-dolichol utilization defect 1 (*MPDU1*) [26], a defect in the flipping of Dol-P-Man across the endoplasmic reticulum membrane was suggested, likely affecting *O*-mannosyl glycosylation. In cases involving mutations in the *TRAPPC11* or *GOSR2* genes [27], the cause of reduced DG glycosylation is unclear. However, because both gene products, trafficking protein particle complex subunit 11 (TRAPPC11) and golgi SNAP receptor complex member 2 (GOSR2), play a role in the structural maintenance and function of the Golgi apparatus, mutations in these genes may change the machinery for DG glycosylation in the Golgi apparatus. These cases may not be considered as genuine DGpathy since the modification of glycoproteins other than DG is also affected (rather, they are likely more severely defective than DG). Elucidation of the molecular pathological mechanisms of CDG associated with DG glycosylation abnormalities will be an important issue in understanding the mechanisms of DG glycosylation.

## 3. DGpathy Model Mice

The first reported, and perhaps the most widely used, DGpathy animal model is *Large*^myd^ mice with spontaneous mutation of the *Large1* gene [28]. *Large*^myd^ mice have structural abnormalities in the brain and eyes, in addition to muscular dystrophy [4,29]. *Large1*-mutant mice also include spontaneously occurring *Large*^vls^ mice and *Large*^enr^ mice resulting from a transgene integration event [30,31]. POMGNT1-knockout (KO) mice, reported by groups in the United States and Japan, have significantly milder muscular lesions than *Large*^myd^ mice [32,33]. POMGNT2-KO mice die within one day of birth [34], and POMT1-KO and fukutin-KO mice suffer embryonic lethality [35,36]. As DG has been shown to be important for the formation and maintenance of a basement membrane called Reichert’s membrane at the embryonic stage of mice [37], POMT1-KO and fukutin-KO mice also exhibit abnormalities in the basement membrane at the embryonic stage. Moreover, conditional KO (cKO) mice have been created to avoid embryonic lethality, and skeletal muscle-, cardiac muscle-, and central nerve-selective fukutin-cKO, POMK-cKO, and POMT2-cKO mice have been reported [24,38,39,40,41,42]. In addition, knock-in (KI) mice with pathogenic mutations found in DGpathy patients have also been created. Transposon insertion mutation in the 3′UTR of *FKTN* is often found in FCMD patients [43], whereas *Fktn*-KI mice with this insertion mutation exhibit no pathological changes because some DG sugar chains remain normal [44]. FKRP-mutant mice were created independently by groups in the United Kingdom and the United States [45,46,47]. This model has been used to reproduce the wide clinical spectrum found in DGpathy patients with FKRP mutations, such as embryonic lethality and muscular dystrophy with/without brain abnormalities depending on the specific point mutation involved. The pathological mechanisms and treatment strategies revealed from studies using these disease-model mice are introduced below.

## 4. Pathological Mechanism of DGpathy

### 4.1. Muscular Dystrophy

In DGpathy, the loss of ligand binding activity of DG due to sugar chain abnormalities is thought to disrupt association with the basement membrane, which reduces the physical stability of the muscle cell membrane, making necrosis more likely to occur [7]. This pathological mechanism suggests that DGpathy patients exhibit muscle pathology similar to those of patients with dystrophin-deficient Duchenne-type muscular dystrophy (DMD). However, some DGpathy patients clearly have more severe disease manifestations than DMD patients, which cannot be explained by only the disruption of association between the basement membrane and cell membrane. Muscle fiber-selective MCK-fukutin-cKO mice present only extremely mild muscular dystrophy, suggesting that there are factors other than susceptibility of muscle fibers to necrosis [38,39]. Muscle progenitor cell-selective Myf5-fukutin-cKO mice exhibit a decreased number of muscle satellite cells and decreased proliferative activity and differentiation activity of muscle progenitor cells with progression of the pathological condition, which results in impaired muscle regeneration [39]. A study using *Large*^myd^ mice reported that abnormalities in the basement membrane environment around satellite cells may also impair muscle regeneration [48]. In addition, embryos of FKRP-mutant mice show a decrease in the number of muscle progenitor cells, muscle fiber diameter, and muscle differentiation capacity [49]. These impairments in muscle development during embryonic stages may also affect postnatal muscle maturation and regenerative capacity. Furthermore, structural and functional abnormalities involving neuromuscular junctions, associated delays in muscle maturation, and changes in the fiber type of regenerating muscles are also thought to be related to the pathological condition [50,51,52].

In addition, a recent study using induced pluripotent stem (iPS) cells derived from FKRP-deficient LGMD2I or WWS patients revealed decreased autophagy and increased apoptosis in myotubes, indicating that alterations in cell homeostasis may be involved in DGpathy pathogenesis [53]. Effective biomarkers of DGpathy, including miRNA and lncRNA, have not been established to date. Metabolomics analysis and miRNA profiling using FKRP mutant mice or DGpathy patient muscle biopsies have been reported [54,55]. The metabolomics study found several pathways potentially associated with pathogenesis such as extracellular matrix remodeling and lipid metabolism. It is hoped that such studies will lead to the discovery of biomarkers and the elucidation of new pathological conditions in the future.

Wood et al. recently reported interesting results [56]. They found that FKRP is involved in sialic acid modification of basement membrane fibronectin and that collagen does not accumulate due to impaired fibronectin glycosylation caused by FKRP abnormalities, leading to basement membrane abnormalities and deterioration of physiological muscle function. Despite a lack of evidence for the enzymatic activity of FKRP in sialic acid modification, functions of FKRP other than ribitol-phosphate modification of DG have been suggested, which may be a clue to explain the diversity of clinical symptoms of DGpathy patients with FKRP mutations.

### 4.2. Central Nervous System Abnormalities

Brain abnormalities in DGpathy patients are diverse, ranging from severe brain malformation and associated mental retardation/refractory epilepsy to average intelligence with little brain malformation [10,11]. Although an association between the type of gene mutation and the severity of the disease has been suggested, the underlying mechanism of the broad clinical spectrum of brain abnormalities in DGpathy patients remains unclear. DG is expressed in the termini of radial glia in the developing cerebral cortex, contributing to maintenance of the glia limitans-basement membrane complex. DG sugar chain abnormalities impair its binding to the basement membrane, disrupting the glia limitans-basement membrane complex [29,57,58]. The protrusion of neuronal cells from the site of basement membrane disruption into the subarachnoid space is thought to be the major pathological mechanism leading to type II lissencephaly [59].

Malformations, such as fusion of cerebral fissures and ectopic cellular infiltration into layer I of the cerebral cortex due to DG sugar chain abnormalities and loss of ligand binding capacity, are observed in *Large*^myd^ mice, POMGNT1-KO mice, POMGNT2-KO mice, and FKRP-mutant mice [4,45,46,60,61,62]. In the developing fetal brain of DGpathy models, radial glia in the cerebrum and Bergmann glia in the cerebellum invade the subarachnoid space, which coincides with the site of basement membrane disruption and disrupts the function and localization of cells that serve as scaffolds for neuronal cell migration. It is notable that very recently, Taniguchi-Ikeda et al. successfully generated three-dimensional brain organoids from FCMD patient-derived iPS cells, which recapitulated abnormal radial glial fiber migration [63]. *Large*^myd^ mice, neural stem cell-selective Emx1-POMT2-cKO mice, and POMGNT1-KO mice also have structural abnormalities of the hippocampal dentate gyrus with loss of pial basement membrane [42,64]. In addition, neurological dysfunction, such as reduced long-term potentiation of hippocampal CA3–CA1 synapses in *Large*^myd^ mice, has also been reported [57].

Sudo et al. performed an analysis focusing on DG sugar chains and the severity of lesions in the fetal brain by using four mouse models of DGpathy with different sugar chain modification status [40]. They found that the severity of brain structural abnormalities differed depending on whether DG sugar chains remained in the glial limitans-basement membrane complex at embryonic day 13.5. Furthermore, they successfully prevented the onset of severe cerebral cortex malformation by introducing a normal gene at embryonic day 12.5. These findings are interesting in considering the clinical diversity of brain abnormalities in DGpathy patients, and these suggest that regulation of DG glycosylation at the fetal stage may be a new treatment strategy for brain abnormalities involved in DGpathy.

### 4.3. Cardiomyopathy

Heart failure, along with respiratory disorders, is the leading cause of death in DGpathy patients. Many FCMD patients, for example, have left ventricular systolic impairment despite the absence of left ventricular hypertrophy and fibrosis is found at autopsy [65]. In addition, dilated cardiomyopathy in fukutin-mutated patients with very mild muscular lesions has been reported [66]. As with skeletal muscle, sugar chain abnormalities are thought to cause impaired association between the basement membrane and cell membrane [67], but cardiomyopathy associated with DGpathy is generally mild in many cases. With regards to model mice, no myocardial lesions or cardiac dysfunction are observed in young (up to 24 weeks old) striated muscle-selective MCK-fukutin-cKO mice. However, despite the absence of cardiac hypertrophy, mice older than 1 year of age exhibit fibrosis in addition to decreased cardiac function, such as chamber dilation during diastole and decreased fractioning shortening, reproducing the cardiac pathology seen in FCMD patients [41]. Moreover, the contractile property of cardiomyocytes isolated from MCK-fukutin-cKO mice is reduced, and intracellular Ca^2+^ handling during excitation-contraction coupling is disrupted. Similarly, mild pathological changes and decreased cardiac function have been reported in FKRP-mutant mice [68].

Interestingly, unaffected young MCK-fukutin-cKO mice do not show the adaptive hypertrophic response to hemodynamic loading, which is observed in normal myocardium, which results in decreased cardiac function and fibrosis, leading to the onset of heart failure [41]. This suggests that DG sugar chains play an important role in the hypertrophic response of cardiomyocytes to hemodynamic changes. The milder myocardial lesions compared to skeletal muscular lesions may be due to the small physiological and structural contribution of DG sugar chains in the myocardium or the presence of a compensatory mechanism. On the other hand, tamoxifen-inducible MCM-fukutin-cKO mice die within approximately one week after Cre-recombination induction [41]. In cardiomyocytes isolated from MCM-fukutin-cKO mice, fragmentation of the Golgi apparatus and microtubule hyperpolymerization along the contractile axis are found to occur, and microtubule hyperpolymerization is thought to act as a physical barrier, causing a decrease in contractile function and inducing severe heart failure. In fact, the administration of microtubule polymerization inhibitors improves cardiac function and survival rate, suggesting that microtubule inhibitors are effective therapeutic candidates for cardiomyopathy seen in muscular dystrophy patients.

## 5. Treatment Methods

### 5.1. Gene Therapy

Because DGpathy is a single-gene disorder, gene therapy is considered a simple treatment strategy. As mentioned above, we showed that vulnerability of muscle fibers triggers the onset of DGpathy and that poor muscle regeneration due to decreased function of muscle progenitor cells is associated with pathological severity and progression, using skeletal muscle-selective fukutin cKO mice [39]. Since glycosylation status in muscle progenitor cells and myoblasts changes during differentiation [21], the expression of glycosyltransferases may also be strictly controlled. Therefore, these cells are not suitable targets for gene therapy that uses promoters constitutively inducing gene expression. On the other hand, selective gene rescue in muscle fibers is expected to suppress myonecrosis that triggers muscle regeneration and disease onset, although it does not improve poor muscle regeneration. In fact, the administration of an adeno-associated viral vector that allow a muscle fiber-selective expression of the *fukutin* gene dramatically ameliorates muscular dystrophy in Myf5-fukutin-cKO mice that exhibit muscle regeneration abnormalities [39]. The effectiveness of gene therapy for DGpathy has also been demonstrated in FKRP-mutant mice and *Large*^myd^ mice [69,70,71]. Dhoke et al. recently reported a gene correction approach with the CRISPR-Cas9 gene editing for FKRP-deficient DGpathy to enable cell therapy [72]. In this study, the authors corrected FKRP mutations in iPS cells derived from FKRP-deficient DGpathy patients using homology-directed repair to knock in the wild-type sequence, differentiated the gene-corrected cells into myogenic progenitors, and then transplanted them in the FKRP-mutant mice. Results showed restoration of glycosylation in engrafted muscles, indicating a potential of the combination of gene editing and iPS cell-derived myogenic cell transplantation as a therapeutic approach in the future.

Interestingly, *B4GALNT2* (previously *GALGT2*) gene therapy is reported to exert therapeutic effects on muscular dystrophy in FKRP-mutant mice [73]. The expression of B4GALNT2, which is a glycosyltransferase, was shown to have a therapeutic effect on various types of muscular dystrophy models, such as dystrophin-deficient and laminin-deficient mice. The exact mechanism underlying this pathological improvement is unknown, but several pathways, such as increased expression of matrix molecules, their receptors, and structural proteins, have been postulated. For FKRP-mutant mice, it is likely its therapeutic effect arises through mechanisms such as improvement in muscle regeneration, rather than by compensating for DG sugar chain abnormalities [73].

A treatment strategy targeting LARGE activity has been attracting attention as a glyco-therapy for many years. Overexpression of the *LARGE* gene increases matriglycan modification and enhances laminin-binding activity [20]. Interestingly, in cells derived from DGpathy patients and model mice with POMT1, POMGNT1, FKTN, or FKRP mutation, overexpression of LARGE enhances laminin-binding activity as long as there is residual activity of the mutated gene product [44,71,74]. Therefore, *LARGE1* gene therapy has attracted attention as a treatment method that does not depend on the type of causative gene. On the other hand, it may have adverse effects due to the constitutively active matriglycan modification in multiple cells. In fact, systemic overexpression of *LARGE1* is known to exacerbate muscular dystrophy [75,76]. Thus, in treatment using the *LARGE1* gene as a therapeutic target, its expression level, target tissues, and intervention method must be strictly considered. In addition, low-molecular-weight compounds, rather than gene therapy, may be a viable option in certain cases. In fact, compounds that enhance matriglycan modification have been identified [77,78], and these, including compounds that enhance *LARGE1* gene expression, are interesting therapeutic candidates. In addition, instead of targeting matriglycan, antibody therapy that connects cell membrane and basement membrane using a bivalent antibody against laminin and DG has been proposed [79].

### 5.2. Pharmacotherapy

Foltz et al. reported that the mTOR pathway is activated in the muscle of fukutin-deficient mice with advanced pathology and that inhibition of the mTOR pathway with rapamycin suppresses muscular lesions such as fibrosis [80]. Binding between DG and matrix proteins is known to be involved not only in the structural maintenance of myocytes, but also in intracellular signal transduction, but DG sugar chain abnormalities may not directly activate the mTOR pathway. Thus, rapamycin may act on fibroblasts to inhibit the synthesis of matrix proteins, resulting in the suppression of fibrosis. Selective estrogen receptor modulators (SERMs) are expected to be effective against muscular dystrophy because of their features such as anti-inflammation, anti-fibrosis, bone loss suppression, and muscle mass gain. Wu et al. conducted long-term administration of two SERMs, tamoxifen and raloxifene, in FKRP-mutant mice, showing improvements in muscle function and muscle pathology [81]. Although the detailed mechanisms of action of both rapamycin and SERMs are yet to be elucidated, they are of interest as disease-modifying drugs for DGpathy. Myostatin is a negative regulator of muscle growth, and myostatin inhibition therapy has been proposed for various diseases associated with muscle loss, such as muscular dystrophy [82]. With regards to DGpathy, a clinical trial of a myostatin inhibitor has been conducted in patients with FKRP mutation, but its effectiveness has not been observed to date [83].

Screening for low molecular-weight compounds effective in the treatment of DGpathy has been performed using FKRP-mutant zebrafish [84], which identified hits including steroids, non-steroidal anti-inflammatory drugs (NSAIDs), antibacterial drugs, and calcium chelators. Steroids have been used clinically in muscular dystrophy patients, and the beneficial effects of glucocorticoid (predonisolone) administration on the pathological condition has been suggested in FKRP-mutant mice [85]. In addition, its combined use with the osteoporosis drug alendronate was shown to increase this therapeutic effect. Interestingly, recovery of DG sugar chains was observed after the administration of predonisolone/alendronate in this study. While the mechanisms involved remain completely unknown, it would be of great interest to be able to control the DG glycosylation pathway, in addition to steroid action such as anti-inflammation. As introduced above, identification of effective therapeutic agents from the results of molecular pathological analysis of disease models and drug screening is expected.

A unique treatment strategy for FCMD based on molecular pathology has been reported. Many FCMD patients have a transposon insertion in the *fukutin* gene [43]. A strong RNA splice-acceptor site exists in this insertion, which activates a cryptic splicing donor site in the protein-encoding final exon, resulting in abnormal splicing of *fukutin* [86]. Administration of antisense nucleotides capable of correcting this splicing abnormality restores the normal function of fukutin in both fukutin KI mice and human patient-derived cells. Because this antisense nucleotide therapy can be applied to almost all FCMD patients, it represents a promising fundamental molecular-targeted therapy, and rapid progress is expected in the future.

### 5.3. Ribitol Supplementation Therapy

**Figure 2:**
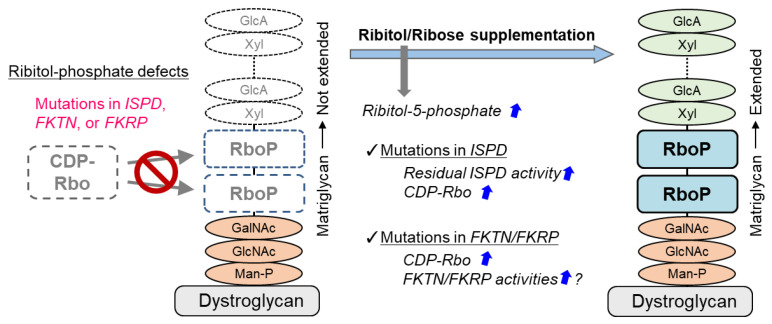
Treatment strategy for ribitol phosphate-deficient DGpathy. DGpathy with ribitol phosphate defects may be treatable. Details are explained in the text.

A treatment strategy for DGpathy that has received the most attention recently is perhaps ribitol supplementation therapy (Figure 2). The donor substrate for fukutin and FKRP, which are ribitol phosphate transferases, is CDP-Rbo, which is produced from ribitol-5-phosphate and CTP by the enzymatic action of ISPD. Although the biosynthetic mechanism of ribitol-5-phosphate has not been understood, the pentose phosphate pathway is thought to be involved [17]. The addition of ribitol and ribose increases the level of intracellular CDP-Rbo in normal cells and wild type mice possibly because they are somehow converted to ribitol-5-phosphate. CDP-Rbo production was shown to increase even if ribose or ribitol was added to fibroblasts derived from patients with ISPD mutations [87]. An increase in the ISPD substrate level likely enhances the enzymatic activity of the mutant protein. However, this effect likely depends on the mutation type, and it is thereby dependent on residual ISPD activity. To overcome this issue, CDP-Rbo replacement therapy has been proposed [15]. This treatment may be applicable to all forms of ISPD mutations, although the efficiency of CDP delivery into cells and its in vivo stability need to be improved.

Interestingly, long-term administration of ribitol to FKRP-mutant (P448L) mice results in restoration of DG glycosylation and therapeutic effects in muscular dystrophy [88]. It is interpreted that the administration of ribitol increases CDP-Rbo production and enhances the enzymatic activity of mutant FKRP thereby improving DG glycosylation. Also, a report suggested that ribitol therapy is effective against the FKRP-L276I mutation, which is most commonly found in patients in Europe and the United States [89]. Ribitol therapy for FKRP mutation may also require residual FKRP enzymatic activity [90]. It is noted that clinical trials testing ribitol (BBP-418) for LGMD2I patients have started in 2021 (NCT04800874). It was recently reported that co-addition with NAD^+^ increased the improvement effect of ribitol or ribose administration on glycosylation of DG [90]. The detailed mechanisms are unknown, but because NAD^+^ is required for metabolic enzymes (oxidoreductases) in the pentose phosphate pathway, CDP-Rbo production may have been increased additively. However, since the administration of NAD^+^ alone does not affect CDP-Rbo levels, its effects may be different from that of CDP-Rbo synthesis. Interestingly, administration of NAD^+^ has an enhancing effect on muscular lesions in DG- or FKRP-deficient zebrafish [91]. It has been suggested that NAD^+^ suppresses muscle degeneration by promoting clustering of basement membrane receptors, such as DG and integrin, and enhancing laminin organization, and independently enhances mitochondrial function. In any case, it should be noted that substrate supplementation therapy has different efficacy depending on the specific type of enzyme mutation. In addition, there is a concern that excessive substrate administration may result in fluctuations involving the metabolic balance in the body, causing adverse effects. In particular, the toxicity of ribitol needs to be verified. On the other hand, ribose is commercially available as a supplement, and the vitamin B3 group that increases levels of NAD^+^ is also commercially available. If such compounds that are structurally simple and found in the living body can be used as therapeutic agents for DGpathy, their benefits will be immeasurable.

## 6. Conclusions

While almost 20 years have passed since the discovery of DGpathy, it has been only approximately 5 years since the elucidation of DGpathy gene function and sugar chain structure. However, a series of studies has had a considerable ripple effect on basic academic fields such as glycobiology. Additionally, combined with pathological studies conducted by researchers in the field of muscle disease biology, those studies have proposed several promising treatment strategies, and some have demonstrated their proof-of-concept at the animal level. Progress in translational research is expected in the future, which will eventually overcome DGpathy.

## Figures and Tables

**Table 1:** DGpathy genes and gene product functions.

DGpathy Genes	Gene Functions
*POMT1*	Protein *O*-mannosyl-transferase as a part of the POMT1/2 complex
*POMT2*	Protein *O*-mannosyl-transferase as a part of the POMT1/2 complex
*POMGNT1*	Protein *O*-mannose β1,2-*N*-acetylglucosaminyltransferase; Core M1 synthesis
*POMGNT2*	Protein *O*-mannose β1,4-*N*-acetylglucosaminyltransferase; Core M3 synthesis
*B3GALNT2*	β1,3-*N*-acetylgalactosaminyltransferase; Core M3 synthesis
*POMK*	Protein *O*-mannose kinase; phosphorylation of Core M3
*FKTN*	Ribitol phosphate transferase; tandem ribitol synthesis
*FKRP*	Ribitol phosphate transferase; tandem ribitol synthesis
*ISPD*/*CRPPA*	CDP-ribitol pyrophosphorylase; synthesis of CDP-ribitol (donor substrate of FKTN/FKRP)
*TMEM5*/ *RXYLT1*	Ribitol-5-phosphate xylosyltransferase; synthesis of linker structure between tandem ribitol and matriglycan
*B4GAT1*	β1,4-Glucuronyltransferase; synthesis of linker structure between tandem ribitol and matriglycan
*LARGE*	Xylosyl- and glucuronyltransferase; matriglycan synthesis
*DAG1*	Dystroglycan
*GMPPB*	GDP-mannose pyrophosphorylase required for the formation of GDP-Man; Dolichol-phosphate-mannose synthesis
*DPM1*	Dolichol-phosphate-mannose synthase; Dolichol-phosphate-mannose synthesis
*DPM2*	Dolichol-phosphate-mannose synthase; Dolichol-phosphate-mannose synthesis
*DPM3*	Dolichol-phosphate-mannose synthase; Dolichol-phosphate-mannose synthesis
*DOLK*	Dolichol kinase required for the formation of dolichol-phosphate; Dolichol-phosphate-mannose synthesis
